# Parasites, parasitoids, and hive products that are potentially deleterious to wild and commercially raised bumble bees (*Bombus* spp.) in North America

**DOI:** 10.26786/1920-7603(2023)710

**Published:** 2023-09-02

**Authors:** Elaine C. Evans, James P. Strange, Ben M. Sadd, Amber D. Tripodi, Laura L. Figueroa, Laurie Davies Adams, Sheila R. Colla, Michelle A. Duennes, David M. Lehmann, Heather Moylett, Leif Richardson, James W. Smith, Tamara A. Smith, Edward M. Spevak, David W. Inouye

**Affiliations:** 1 Department of Entomology, University of Minnesota, Saint Paul, MN 55108 USA; 2 Department of Entomology, The Ohio State University, Columbus, OH 43214; 3 School of Biological Sciences, Illinois State University, Normal, IL 61790, USA; 4 Unaffiliated, Raleigh, North Carolina, 27604 , USA; 5 Department of Environmental Conservation, University of Massachusetts, Amherst, Amherst, MA, 01003, US; 6 Department of Entomology, Cornell University, Ithaca, NY, 14850, USA; 7 Pollinator Partnership, 600 Montgomery, Suite 440, San Francisco, CA 94111; 8 SRC: Faculty of Environmental and Urban Change, York University, Toronto, ON, Canada; 9 Department of Biology, Saint Vincent College, Latrobe, PA 15650; 10 Center for Public Health and Environmental Assessment (CPHEA), Health and Environmental Effects Assessment Division, Integrated Health Assessment Branch, US - Environmental Protection Agency, Research Triangle Park, NC, 27711, USA; 11 Unaffiliated, Garner, North Carolina, 27529, USA; 12 The Xerces Society for Invertebrate Conservation, 628 NE Broadway, Suite 20, Portland, OR 97232-1324, USA; 13 Retired, USDA-Animal and Plant Health Inspection Service, Raleigh, NC 27526, USA; 14 U.S. Fish & Wildlife Service, Minnesota- Wisconsin Ecological Services Field Office, 3815 American Boulevard East, Bloomington, MN 55425; 15 Center for Native Pollinator Conservation, Saint Louis Zoo, One Government Drive, St. Louis, MO 63110, USA; 16 Department of Biology, University of Maryland, College Park, MD 20742, and Rocky Mountain Biological Laboratory, PO Box 510, Crested Butte, CO 81224, USA

**Keywords:** *Bombus*, bumble bee, symbionts, parasites, pathogens

## Abstract

Bumble bees are important pollinators for a great diversity of wild and cultivated plants, and in many parts of the world certain species have been found to be in decline, gone locally extinct, or even globally extinct. A large number of symbionts live on, in, or with these social bees. We give an overview of what is known about bumble bee ecto-symbionts and parasitoids. We provide information on assessment of risks posed by select bumble bee symbionts and methods for their detection, quantification, and control. In addition, we assess honey bee hive products such as pollen and wax that are used in commercial bumble bee production, and highlight key risks and knowledge gaps. Knowledge of these potential threats to native pollinators is important and they need to be managed in the context of national and international commercial trade in bumble bees to prevent pest introduction and pathogen spillover that can threaten native bees.

## Introduction

Bumble bees (*Bombus* spp.) are widely distributed primarily in temperate and alpine regions of the world, are exceptionally good pollinators of many cultivated and wild plant species, and are now the focus of a well-developed and growing commercial market involving both national and international trade (Goulson 2009). As is true for many species, they have a large number of symbionts, which bridge the range from innocuous to lethal in their effects on their hosts. We have reviewed elsewhere the diversity of endosymbionts, including viruses, bacteria, protozoans, fungi, and nematodes, but excluding tracheal mites (Figueroa et al. 2023), and consider here the smaller number of ectosymbionts, parasitoids, and the commerce in pollen and wax that could impact both wild and domesticated colonies of bumble bees (*e.g*., *B. terrestris* in Europe, *B. impatiens* in North America and *B. huntii* in western Canada).

## 1. Ectosymbionts

### Acarines

There are at least 91 mites associated with bumble bees (Klimov et al. 2016; Klimov et al. 2017), yet most that are found on the host’s exterior are considered to be harmless nest commensals (Table S1). Mites are most often found on queens, with one study finding mites on 74% of queens, with lower frequency on males (37%) and workers (27%) (Haas et al. 2019). The mites found externally on spring queens are not known to survive in the commercial rearing environment (Velthuis & van Doorn 2006). *Scutacarus acarorum*, an inquiline of bumble bee nests known to feed primarily on fungus (Jagersbacher-Baumann & Ebermann 2013) has incorrectly been described as an occasional parasite of bumble bee larvae (Jagersbacher-Baumann 2015). Other bumble bee-associated mites that are thought to have non-parasitic life histories include *Kunzia americana, K. affinis, Parasitellus* (formerly *Parasitus*) spp., *Proctolaelaps longisetosus,* and *P. bombophilus* (Delfinado & Baker 1976; Eickwort 1990; Goldblatt & Fell 1987; Richards & Richards 1976). Most of these mites are thought to be scavengers or fungivores within nests rather than associated with individual bees, although some are predatory and may benefit the bumble bees by consuming nest pests (Eickwort 1990). Others have an uncertain status in nests. *Pneumolaelaps* species seem to be obligate specialists in bumble bee nests, and although they have been observed feeding on injured bees, they might be best classified as kleptoparasites that consume only the freshly collected pollen intended for larvae (Hunter & Husband 1973; Royce & Krantz 1989). While many mite species are expected to be phoretic or commensalistic (Houck 1993), the exact incidence across different life stages and different species of bumble bees is not well documented. On the whole, the ecologies of mites are understudied, and completely unknown for some bumble bee associates, like the *Cerophagus* spp. (O’Connor 1992).

One mite, *Locustacarus buchneri*, is an endoparasite, but included here with other acarines for consistency. This bumble bee tracheal mite is an internal parasite inhabiting the airways and abdominal air sacs of adult bees (Husband & Sinha 1970). It has been reported to lead to lethargy and reduced foraging (Husband and Sinha 1970) and infected male bumble bees brought into the laboratory have reduced longevity (Otterstatter & Whidden 2004). In North America, it seems to be more common in early-emerging species, such as *B. bimaculatus, B. perplexus,* and *B. vagans* (Macfarlane et al. 1995), but not all early-season species are affected (*e.g., B. mixtus* in Canada (Otterstatter and Whidden 2004). Bees are infected as 3rd instar larvae, female mites overwinter within new queens (gynes), and populations build quickly and spread throughout the colony after establishment in the spring (Yoneda et al. 2008a). Colonies infected with *L. buchneri* have been purchased from commercial sources (Otterstatter et al. 2005; Yoneda et al. 2008b), and there has been concern that commercial trafficking of bumble bees will carry this parasite into novel hosts (Goka et al. 2001). The mite is widely distributed in the Northern Hemisphere, and has been found in Argentina (Plischuk et al. 2011), and in New Zealand, where it was introduced along with its bumble bee hosts (Macfarlane 1975). Rearing companies have been made aware of the need to control this mite (Goka et al. 2001). At present, the consensus is that mites seem well-controlled in colonies sold commercially (Meeus et al. 2011), and in European surveys, even phoretic mites were absent until colonies were deployed in the field (Rożej et al. 2012).

### Dipterans

*Apocephalus borealis* is a parasitoid phorid fly widely distributed throughout North America (Brown 1993). Females oviposit one or more eggs into the body of the host and larvae feed upon the host’s tissues until pupation. Mature larvae leave the host’s body between the head and pronotum prior to pupation, often decapitating the host in the process (Core et al. 2012) , and they may reduce worker lifespans by up to 70% (Otterstatter et al. 2002). Although there are few host records for this species, it has been recorded as a parasite of not only bumble bees (*B. bifarius, B. californicus, B. flavifrons, B. melanopygus, B. occidentalis* and *B. vosnesenskii*), but also black widow spiders (*Latrodectus mactans*), yellowjacket wasps (*Vespula* spp.), and most recently (but less commonly than bumble bees), honey bees (*A. mellifera*) (Brown 1993; Core et al. 2012; Otterstatter et al. 2002). In honey bees, phorid parasitism causes aberrant behavior, such as flying at night and nest abandonment (Core et al. 2012). Parasitism of bees seems seasonal, with peak rates observed in late summer (Core et al. 2012; Otterstatter et al. 2002). In addition, both adult and larval *Apocephalus borealis* tested positive for *Vairimorpha ceranae* and Deformed Wing Virus using molecular tests, suggesting that the flies have the potential to vector these pathogens among species (Core et al. 2012).

Bumble bees are also prey to parasitism by conopid flies. As with phorid parasitoids, conopid females oviposit into adult bees, which they attack while the bees are foraging (Schmid-Hempel & Stauffer 1998), and their larvae are endoparasitoids. Although more than one egg may be laid, only one larva will advance to pupation in a single host (Schmid-Hempel & Schmid-Hempel 1989). Larvae initially consume hemolymph, then move to the fat body, ovaries, and other vital organs, killing the host as they mature (Abdalla et al. 2014). Pupation takes place inside the dead host, and some bumble bee hosts have been shown to bury themselves in soil just prior to the parasitoid’s pupation (Malfi et al. 2014); this behavior is of little consequence to the bee, which is about to die, but is assumed to improve survival and fitness of the parasitoids, which must be inducing it. Conopid parasites have also been shown to alter behavior of infected workers, causing them to spend the night outside of the colony, where cooler temperatures may retard the parasitoid development and therefore prolong the worker’s lifespan (Müller & Schmid-Hempel 1993). Infected bees may also alter their choice of flowers while foraging (Schmid-Hempel & Schmid-Hempel 1990), and their choice of pollen or nectar (Schmid-Hempel & Durrer 1991; Schmid-Hempel & Schmid-Hempel 1991).

Little is known about the host ranges of these flies, but in North America at least five species have been documented to attack *Bombus* spp. (Additional genera parasitize European bumble bees (e.g., *Conops*, *Myopa* and *Sicus*; Smith 1969). Most conopid parasitoids of *Bombus* in North America are in the genus *Physocephala*. One record of *Zodion oblique fasciatum* from a *B. auricomus* host (Frison 1917) was apparently misidentified (Frison 1926; Müller & Schmid-Hempel 1993), but there are two additional records of *Zodion* sp. from Canada that have not been verified (MacFarlane & Pengelly 1974). *Physocephala burgessi* has been found parasitizing *B. pensylvanicus sonorus*; *P. marginata* has been recovered from *B. fervidus* and *B. nevadensis*; *P. sagittaria* has been recorded in *B. auricomus* and *B. pensylvanicus; P. texana* has been found parasitizing *B. bifarius*, *B. californicus*, *B. flavifrons*, and *B. occidentalis*; *P. tibialis* has been recovered from *B. bimaculatus*, *B. griseocollis*, and *B. impatiens* (Freeman 1966; Gibson et al. 2016; Malfi et al. 2014; Malfi & Roulston 2014). *Physocephala* are not restricted to bumble bee hosts, however. *Physocephala texana* has been recorded parasitizing honey bees (*A. mellifera*), alkali bees (*Nomia melanderi*), and sand wasps (*Bembix* spp.), and *P. marginata* has been recovered from honey bees and a leafcutter bee (*Megachile mendica*) as well (Gibson et al. 2016; Parsons 1948). A modeling study, based on field-data of foraging and risk of conopid parasitism, suggested that conopids may not dramatically affect reproductive output of bumble bee colonies when resources are abundant, but may interact with low resource conditions to the significant detriment of colony demographic performance (Malfi et al. 2018). Infection rates can be high; a study of Swiss bumble bees found on average 13.2% of workers and 7.1% of males contained a conopid pupa, with a maximum of 46.7% of workers at one site (Schmid-Hempel et al. 1990). A study in Canada found variation among four *Bombus* species, with a range from 0 – 15% of workers; unparasitized workers did not survive significantly longer than those parasitized by conopids (Otterstatter et al. 2002).

Sarcophagid flies have been infrequently reported as parasites of bumble bee adults and larvae, but as most are primarily scavengers; their status as true parasitoids has been questioned (Dahlem & Downes 1996). North American records of sarcophagid flies thought to have parasitized bumble bees include *Boettcheria litorosa* (also as *Sarcophaga litorosa*)*, Liosarcophaga sarracenioides* (as *Sarcophaga sarracenioides or S. tuberosa sarracenioides*)*, Brachicoma* spp. (*Brachycoma* [sic] *sarcophagina,*), and *Helicobia morionella* (also as *Sarcophaga morionella*) (Frison 1926; Macfarlane et al. 1995; MacFarlane & Pengelley 1977; MacFarlane & Pengelly 1974; Ryckman 1953; Stone 1965). In Ontario, a collection of 385 wild adult bumble bees yielded 3.3% with an endoparasitic sarcophagid larva (MacFarlane & Pengelley 1977). In a captive *B. fervidus* nest, 78% of the cocoons held immatures parasitized by sarcophagid flies, but the parasitic nature of these is less certain (MacFarlane & Pengelley 1977). Frison and Plath both experienced large numbers of Sarcophagids in their captive rearing experiments (Townsend 1935), but very little has been (Ryckman 1953) on the relationship between the flies and bumble bees in recent years, and outbreaks have not been reported in modern rearing facilities. Ryckman (1953) reported rearing *Boettcharia litorosa* and *H. morionella* from adult bumble bees, but there have not been more recent reports of this relationship. *Helicobia morionella* are more commonly reported as facultative parasitoids of gastropods (Coupland & Barker 2004; Stegmaier 1972). Members of the Sarcophagid tribe Miltogrammini are associated with Hymenoptera nests, and primarily considered to be kleptoparasites who feed and develop on the provisions provided to brood (Shewell 1989). One European species in this tribe, *Senotainia tricuspis,* has been recorded as an endoparasite of bumble bees, but it is more commonly associated with honey bees (Bailey & Ball 1991). Larvae of the bumble bee mimic syrphid fly *Volucella bombylans* have also been recorded as pests of weak nests, but these organisms are scavengers and are not thought to feed on healthy larvae (Gabritschevsky 1926; Hobbs 1967; Monfared et al. 2013). Because of the mechanisms by which most dipteran parasites of bumble bees locate and parasitize the hosts, the risk of dipterans in rearing facilities is relatively low.

### Hymenopterans

Braconid wasps in the genus *Syntretus* are known as parasites of adult queen, worker and male bumble bees in Europe (Alford 1968; Schmid-Hempel et al. 1990). Although less work has been conducted on wasp parasitoids of bumble bees in North America, 2% of spring-caught queens were parasitized by wasps assumed to be *Syntretus* in Virginia (Goldblatt & Fell 1984), and 3% of *B. vosnesenskii* queens from the West were parasitized with wasp larvae assumed to be *S. splendidus* (Mullins et al. 2019). *Syntretus* wasps oviposit in adult bumble bee hosts while the bees are foraging or resting away from the nest, depositing multiple eggs (mean number of wasps per bee = 23.2) into the membrane between head and prothorax (*i.e*., the first segment of the mesosoma) (Alford 1968). Larvae live in the host for three to four weeks, before exiting the host as fifth-instar larvae via the membrane between the second and third metasomal segments. Successful pupation seems to depend on the presence of soil (Alford 1968), thus these insects are unlikely to establish as pests of captive-reared bumble bees. In England, *Syntretus* parasitization occurs in late May and early June (Alford 1968), suggesting that early-emerging bumble bees may avoid this threat. Parasitization of queens is likely to have the greatest impact on bumble bee populations. The ovaries of parasitized queens atrophy and such queens will eventually stop laying eggs, and nests with parasitized queens may be characterized by having pupae but no new brood (Alford 1968). About 7% of wild-caught *B. pratorum* queens in Ireland were infected with *Syntreus,* and all died before initiating colonies (Rutrecht and Brown 2008). However, parasitized workers continue to forage until shortly before their deaths, suggesting that parasitization of this caste has little effect on the growth and health of the colony (Alford 1968).

Bumble bees are also vulnerable to parasitization by Eulophid wasps in the genus *Melittobia*. Unlike *Syntretus,* which are parasitoids (endoparasites) of adult hosts, the *Melittobia* are idiobiont ectoparasites of immature stages (Dahms 1984b; González et al. 2004). Prior to oviposition on the exterior of the host’s cuticle, *Melittobia* females pierce the cuticle, subduing the host, providing the adult wasp with food in the form of hemolymph, and in some cases, inhibiting the development of the host (González et al. 2004). In *B. terrestris, Melittobia* can only develop on pupae and prepupae (Kwon et al. 2012b). These wasps have a high reproductive capacity, with 200–600 offspring reared on each host (de Wael et al. 1993; de Wael et al. 1995). Fecundity with *B. terrestris* hosts averaged about 48 per mated female wasp under experimental conditions (Kwon et al. 2012a). The *Melittobia* have a wide host range, particularly in the aculeate Hymenoptera and including many species of commercially reared bees: bumble bees, honey bees, and the alfalfa leafcutter bee, *Megachile rotundata* (Dahms 1984b). With such high fecundity and six to eight generations per year, *Melittobia* infestations can greatly impact colony health (de Wael et al. 1995). Infestations of *Melittobia* have caused economic damage in rearing facilities of both leaf cutting bees and bumble bees (Dahms 1984b; de Wael et al. 1995; Kwon et al. 2012a; Nørgaard Holm & Skou 1972).

Due to their wide host range, small size and cryptic habits, wasps in this genus are not only found in the wild (Gekière et al. 2022), but are particularly susceptible to anthropogenic introductions through commercial trade, and this has been reported for two species, *M. acasta* and *M. australica* (Matthews et al. 2009). Populations of *Melittobia* spp. can increase rapidly in artificial rearing conditions due to their gregarious nature, their cryptic habit of remaining on pupal hosts inside of sealed cells, and the rapid development time of the parasite, all which can result in severe damage to a colony and ultimately colony failure (González et al. 2004; Kwon et al. 2012b; Matthews et al. 2009). *Melittobia* are difficult to identify to species and may have wide host ranges, thus many parasite-host records are likely to be inaccurate (Dahms 1984b). Some *M. chalybii* records, including those in North American bumble bees, are likely mis-identified and should be attributed to *M. acasta,* but it is generally accepted that this parasite can develop on a wide range of hosts, at least under laboratory conditions (González & Matthews 2005; González et al. 2004; Husband & Brown 1976; LaSalle 1994). Other records may be of *Melittobia* as a hyperparasitoid, parasitizing other parasitic insects inhabiting bumble bee nests, such as flies (*e.g.,* sarcophagid pupa in *B. vagans* nest (Husband & Brown 1976) or even parasitizing moths in nests. Further inquiry and better taxonomic treatment are necessary to clarify host-parasite relationships in this group (Matthews et al. 2009; Whitfield & Cameron 1993).

Congeners of bumble bees of the subgenus *Psithyrus* are obligate social parasites of bumble bees, with about 30 species worldwide (Williams 2008). They have evolved a number of morphological, social, and behavioral adaptations that reflect their social parasitism, with the loss of corbiculae, an enhanced stinging apparatus, thicker integument, and the loss of a worker caste the most prominent characteristics that distinguish this group (Plath 1922). Female *Psithyrus* invade a nest, kill or dominate the rightful queen, and use the food-gathering and nursing labor of the usurped queen’s workers to rear their own offspring. Many *Psithyrus* are host-specific, occupying the nests of one or a few host bumble bee species (Williams 2008). This host specificity is additionally supported by evidence that some parasites share chemical profiles of their host species that may allow them to overcome host defenses (Martin et al. 2010). Once colonies are deployed in the field, they may come under attack by *Psithyrus* invaders, but these social parasites would not be an issue in captive rearing (Strange et al. 2014). The *Psithyrus* are susceptible to the same parasites as their social cousins *(e.g., S. bombi*, McCorquodale et al. 1998), and may vector some of these into nests as they attempt to invade. Recently, Koch et al. (2021) demonstrated that *Psithyrus* invasions can be prevented by use of a fabricated plastic excluder affixed to the nest entrance, providing protection for field-deployed colonies. Their mode of parasitism, however, makes them highly unlikely to impact rearing facilities or be spread during commercial distribution.

Incidence of parasitism by *Psithyrus* can be high. A study in the UK found that up to 92% of *Bombus terrestris* nests had been invaded. Koch et al. (2021) studied 16 field-deployed colonies of *B. huntii* in two study sites in northern Utah, and with 12 days of deployment 13 of the colonies had at least one *Bombus* (*Psithyrus*) female. Carvell et al. (2008) placed experimental colonies of *B. terrestris* into agricultural fields and found that 38 of the 48 colonies (79%) were invaded by 129 *B*. (*Psithyrus*) *vestalis* females.

### Coleopterans

The invasive small hive beetle, *Aethina tumida* (Nitidulidae), originating from sub-Saharan Africa, is a relatively recently arrived pest of honey bee hives in North America that has the potential to cause destruction to bumble bee colonies as well (Ambrose et al. 2000). The beetles feed on wax, pollen, honey, eggs, and larvae, and can foul food stores through fermentation by associated yeasts (Cuthbertson et al. 2013). Small hive beetles are capable fliers and may disperse over several kilometers (Neumann & Elzen 2004). They can locate bumble bee colonies in field conditions and are attracted to both worker and pollen odors (Spiewok & Neumann 2006). Experimentally infested bumble bee colonies sustained large amounts of damage to the comb and had fewer live bees than a control, indicating that small hive beetle infestation can be devastating to colonies (Ambrose et al. 2000). Bumble bees do show defensive behaviors that help thwart the establishment of small hive beetles within colonies, including egg removal and stinging larvae to death (Hoffmann et al. 2008), but the beetles are cryptic and oviposit in crevices that are often out of the reach of their host bees (Cuthbertson et al. 2013). Because the larvae require soil in which to pupate (Cuthbertson et al. 2013), there is little chance of the beetle becoming a pest in most rearing facilities, but they may pose issues once colonies are deployed in the field (Spiewok & Neumann 2006). The beetle may also vector deformed wing virus (DWV) among colonies, since the virus has been shown to replicate in the beetle (Eyer et al. 2009).

Beetles in the genus *Antherophagus* (Cryptophagidae) are phoretic on bumble bees, hitching a ride back to the nest by attaching themselves to the mouthparts or leg of the foraging bee (Chavarria 1994; Parks 2016; Wheeler 1919). Once back in the nest, the beetles feed and rear their young on nest detritus and are not thought to be detrimental to the colony (Frison 1921). Five species are known from North America, but the genus is widespread, also occurring in South America, Europe, and Asia (Bousquet 1989). Because bees encounter these beetles while free foraging on flowers, and the beetles are merely nest scavengers, they are not presumed to be an issue in commercial rearing.

Hobbs et al. (1962), in a study of 355 bumble bee nests in a two-year study in the foothills of the Canadian Rocky Mountains, found a single larva of the checkered beetle *Trichodes ornatus* in each of five colonies, “apparently eating bumble bee larvae and pupae”. There do not seem to be other reports of this beetle, so it seems unlikely to be a significant problem.

### Lepidopterans

A number of moths in the family Pyralidae are known as pests of bumble bee nests, targeting nest products including wax and pollen, and in some cases, bee larvae. The bee moth *Aphomia sociella* originates from Europe, but is now adventive and widespread throughout North America, specializing on the nests of the aculeate Hymenoptera (Solis & Metz 2008). Infestations by this moth can be devastating to bumble bee nests, as the larvae destroy the comb and consume the brood (Frison 1926; Goulson et al. 2002). An experimental study in the United Kingdom found mean numbers per nest ranging from 2.89 (farms with conservation measures in place) to 77.2 (nests in gardens) (Goulson et al. 2002). Although it has been described as a specialist on bumble bees (Goulson et al. 2002), thriving populations of the moth have been discovered in the nests of Vespid wasps, as well as in mouse and bird nests (Solis & Metz 2008). Aboveground, artificial bumble bee nests may be more easily located by the moth than natural, subterranean ones (Goulson et al. 2002). *Vitula edmandsii,* the American wax moth, may also be an occasional pest of honey bee hive products (Milum 1953). In a mixed apiary with both honey bee and bumble bee colonies, most bumble bee nests were infested with *V. edmandsii* but no honey bee hives contained this pest (Whitfield & Cameron 1993). The larvae of *V. edmandsii* feed upon wax, pollen and other nest materials, but are not known to feed directly upon living larvae (Frison 1926). Its western counterpart, the driedfruit moth *V. serratilineella,* is also known as a pest of *Megachile rotundata,* but because these two moth species have often been considered as one species, it is difficult to discern whether *V. serratilineella* has been associated with bumble bees (Richards 1984; Sattler 1988; Scholtens & Solis 2015).

The greater wax moth *Galleria mellonella* is a well-known pest in honey bee apiaries. Although the greater wax moth has been successfully reared on bumble bee nests (Oertel 1963) and found in field-deployed colonies of *B. impatiens*, bumble bee nests remained free of this pest even when placed in an apiary containing heavily infested honey bee hives (Whitfield & Cameron 1993). This pest can be quite destructive in bumble bee colonies, and heavy infestations can lead to rapid colony declines. The lesser wax moth *Achroia grisella* is a similar pest in honey bee hives, but has not been reported in bumble bee colonies (Milum 1940) and seems to be an issue only in very weak honey bee hives (Williams 1997). The invasive Indian meal moth *Plodia interpunctella* is a stored product pest with worldwide distribution (Williams 1997). With six to eight generations per year, populations of this pest can be quite large, and are highly destructive to colonies in captive rearing facilities (An et al. 2007). Unlike the wax moths discussed previously, the Indian meal moth does not feed on wax, but rather develops on high-protein pollen stores and dead brood and adults (Williams 1997). Moth eggs are sometimes transported into rearing facilities on pollen acquired from honey bees (Kwon et al. 2003). The Mediterranean flour moth, *Ephestia kuehniella,* is a similar pyralid with a worldwide distribution, but it is thought to feed only on pollen provisions in the nest (Milum 1940; Schmid-Hempel 2001).

## 2. Detection, identification, and quantification

### Acarines

Tracheal mite presence is determined through visual examination of the metasomal air sacs under a dissecting microscope (Kissinger et al. 2011; Otterstatter & Whidden 2004). Adult females are nearly spherical, about 450–550 μm across and are the most readily detectable stage, although eggs, males, and larviform females are typically 50–200 μm and usually apparent at low magnification as well (Husband & Sinha 1970). Primers have also been developed for PCR-based detection of tracheal mites (Arismendi et al. 2016; Goka et al. 2001), but there is a need for additional morphological keys to identify species. A useful resource for identification is http://idtools.org/id/mites/beemites/about_tool.php.

Mites on the exterior of bumble bees are not thought to pose a problem but can be detected upon visual examination of the thorax, propodeum, and tergites under low magnification (Kissinger et al. 2011). They can be common, although mites are less common on queens that have founded a nest than those still searching for a nest site, which supports the notion that the mites are more closely associated with nest materials than the bees themselves (Sarro et al. 2022). A survey of 11 *Bombus* species in 15 sites in Ontario, Canada turned up 33 mite species, almost half of which are obligate to bumble bees, although not to particular species (Haas et al. 2019). Queens had the highest incidence (perhaps related to their longevity, or larger body size), followed by males and then workers. The abundance and species richness of mites increased with local bee abundance. Surveys for mites in other bumble bee communities would be useful. A Berlese (Tullgren) funnel might prove useful for collecting mites from nest material.

### Dipterans

Detection of dipteran parasitoids has primarily occurred via visual techniques during dissection, but some can be reared to adulthood if allowed to remain in the body cavity while the flies complete their development (conopids: several months; phorids: 3 weeks: (Otterstatter et al. 2002). Second- and third-instar conopid larvae and pupae can be detected in the metasomal cavity of host bees without magnification due to the large size of the larvae (Malfi & Roulston 2014) and are easily located attached to a metasomal airsac. Typically, however, dipteran larvae are detected during dissection at low magnification (10–40X) to ensure detection of early first-instar larvae, which are smaller and free-ranging in the metasomal haemocoel. Dipteran endoparasites must maintain a connection to the tracheal system of the host bee for respiration, so they are often associated with the metasomal air sacs. Infection rates can be high in some bumble bee populations. Canadian populations had rates of parasitism by phorid flies as high as 20%, although there were significant differences between workers and males, and among species (Otterstatter et al. 2002). Conopid fly parasitism in the same populations was a little lower, with a range of 5–20% typical of the four host species (Otterstatter et al. 2002). A European study found similar rates of parasitism by conopids; on average, 13.2% of all workers and 7.1% of all males contained the puparium of a conopid (Schmid-Hempel & Schmid-Hempel 1990). Although conopid parasitism may not significantly affect lifespan of workers, presence of phorid parasitoids strongly reduced survivorship (Otterstatter et al. 2002).

Identification of dipteran larvae can be challenging for non-specialists, and there are few keys available that can allow for genus or species identification, although family-level identification is relatively simple (McAlpine et al. 1981). Conopid larvae, pupae, and eggs, as well as adults, can be identified to genus using the keys developed by Smith (1987). However, many genera have been added to the family since the development of the larval key in the 1960s. Many adult species of conopids in North America can be identified by the keys of Camras (1996; 1957). Adult phorids can be identified to genus with the key of (Peterson 1987), and species within *Apocephalus* (*Mesophora*) can be identified with the key by Brown (1993). Phorid-specific PCR primers have been developed to detect molecularly internal parasites of bees. Detection of dipteran nest pests and ectoparasites of larval bumble bees, such as the Sarcophagids, would require inspection of the nests and opening nest cells. Family-level identification can be conducted with the adult and larval keys presented in McAlpine (1981), although lower-level classification would require specialized keys.

### Hymenopterans

*Syntretus* wasps can be detected through dissection of adult bees to observe larvae or rearing larvae to adulthood in the carcass of adult hosts. The wasp larvae range in length from 1.8 to 4.3 mm, with the pupae measuring 2.2–3.1 mm long (Alford 1968). Adult wasps found in and around bumble bee nests can be identified to genus using the key of Wharton et al. (1997). Little work has been conducted on this genus in North America, therefore if found, identification to species is unlikely.

*Melittobia* wasps are small (1.0–1.5 mm) and the larvae develop cryptically within the pupal cells of their bumble bee hosts. Therefore, nest inspections using microscopy are generally used for detection, although simple visual inspection is adequate when large outbreaks of the wasps occur and adults are flying (Matthews et al. 2009). A key to genera of the subfamily Tetrastichinae and a list of North America species are available (LaSalle 1994), but *Bombus* are notably absent from the accompanying appendix of host lists. Keys for separating species of *Melittobia* are provided by Dahms (1984a). Identification to species is somewhat possible with adult wasps, particularly males, although there has been much taxonomic confusion in the genus and expert identification is warranted.

The nest parasite bumble bee species within the *Bombus* subgenus *Psithyrus* can easily be identified from adults using subgenus- and species-level keys for bumble bees (Koch et al. 2012; Mitchell 1962; Thorp et al. 1983; Williams et al. 2014).

### Coleopterans

Small hive beetles and *Antherophagus* beetles can be detected upon visual inspection of nests and nest debris. Descriptions of the distinguishing features of all life stages of small hive beetles may be found in Neumann (2013), along with molecular identification, nest inspection, and trapping techniques that can be easily modified for screening bumble bee colonies. Identification of *Antherophagus* to genus can be achieved with the key included in Bousquet (1989), but identification to species is unlikely with existing keys.

### Lepidopterans

Nest-fouling moths can be detected upon visual inspection of nests and rearing facilities and through trap monitoring. Multiple means of monitoring the stored-product pest, *P. interpunctella,* are available, including sticky traps with and without pheromone attractants (Mohandass et al. 2007) and UV light traps. Once established, moths destroy the nest entirely and thus early detection is essential for maintaining colony health (Kwon et al. 2003). There are nearly 5,000 species of pyralid moths and identification to species can be challenging (Solis 2007). Adults of *Aphomia* spp. in North America can be identified with keys in Solis and Metz (2008). The larvae of *Ephestia kuehniella* and *P. interpunctella,* can be identified with the key provided by Solis (2006).

## 3. Hive products and associated risks

### Pollen

Pollen, the primary food for the development of bee larvae, can be a source of exposure to pathogens and pesticides for commercially raised bumble bees. Pollen is frequently contaminated with pathogens (Chen et al. 2006; Gilliam et al. 1988; Higes et al. 2008) and detritus, and may be contaminated with pesticides or other environmental contaminants (Chauzat et al. 2006; Mullin et al. 2010). Recent work has demonstrated the potential role of pollen in moving pathogens from species to species (Graystock et al. 2015; Pereira et al. 2019; Singh et al. 2010), for instance via honey bee pollen collected and used for feeding captive bumble bee colonies. There are no regulations in place governing sanitary practices associated with use of pollen by commercial bumble bee rearing facilities despite the acknowledged threat of pollen in spreading pathogens within and among species (Gilliam et al. 1988; Graystock et al. 2016) and the fact that more than two-hundred tons of honey bee-collected pollen are used annually for bumble bee rearing worldwide (Velthuis & van Doorn 2006).

Several treatments to reduce the spread of pathogens through pollen have been investigated including irradiation (Álvarez Hidalgo et al. 2020; Graystock et al. 2015; Graystock et al. 2016; Meeus et al. 2014; Yook et al. 1998), ozone (Graystock et al. 2016; Yook et al. 1998), pulsed light (Naughton et al. 2017) and ethylene oxide fumigation (Strange et al., in review). Irradiation of pollen at levels from 5 kGy to 16.9 kGy has been shown to eliminate or reduce many pathogens and their infectivity. At lower levels (5 kGy to 7.5 kGy), fungi, coliform and aerobic bacteria, yeasts, and molds were not detected after irradiation (Álvarez Hidalgo et al. 2020), with little effect on pollen nutrition or structure (Yook et al. 1998). At higher levels of irradiation (16.9 kGy), Deformed Wing virus, Israeli Acute Paralysis virus, Sacbrood virus, and *Vairimorpha ceranae* were all removed, while *Crithidia bombi*, *Ascosphaera,* Black Queen Cell virus, and Chronic Bee Paralysis virus were only partly inactivated (Graystock et al. 2016; Simone-Finstrom et al. 2018). *Apicystis bombi* remained infectious after irradiation but infections were reduced by about half (Graystock et al. 2016). These results show promise to reduce negative impacts on bumble bees with these pollen treatments, but there are concerns about potential adverse effects on the nutritional value of irradiated pollen (Graystock et al. 2016; Meeus et al. 2014) and potential negative effects on the gut microbiome (Meeus et al. 2014, Klinger et al. 2019). Notwithstanding, some commercial rearing facilities routinely use irradiated pollen with no known negative effects on bumble bee rearing or performance (Graystock et al. 2016; Meeus et al. 2014).

Other possible pollen treatments to reduce pathogens in pollen include ozone (Graystock et al. 2016; Yook et al. 1998) and pulsed light treatments (Naughton et al. 2017). Compared to irradiation, ozone treatment was deemed less effective (Graystock et al. 2016; Yook et al. 1998), which may be related to the poor distribution of ozone within the pollen samples. Pulsed light was shown to be effective at inactivating *Crithidia bombi* in pollen samples in a single study (Naughton et al. 2017). Strange et al. (in review) demonstrate the efficacy of ethylene oxide fumigation to kill fungal, bacterial, and viral pathogens in pollen, with results equal to or better than irradiation or ozone fumigation. Further, ethylene oxide showed no negative impacts on pollen consumption by bumble bees, nor did it impact colony growth. However, more work is warranted for all sterilization techniques to identify treatment conditions that effectively eliminate pathogens while maintaining nutritional content.

Another potential solution to issues associated with both pathogen and pesticide contamination of pollen is the development of a commercially available pollen substitute. Commercial bumble bee rearing facilities and research programs alike could benefit from a pathogen- and pesticide-free pollen substitute. Use of a pollen substitute would eliminate a source of experimental variability (*i.e*., varying composition of pollen batches). While pollen substitutes for honey bees are well established (Haydak & Dietz 1965; Mattila & Otis 2006), to date, only two publications have investigated potential pollen substitutes for bumble bees (Bortolotti et al. 2020; Graystock et al. 2016). While results from these studies demonstrate significant progress, much work is needed before a suitable pollen substitute will be available for widespread use.

### Wax

Wax is integral in the structure of bumble bee colonies, being produced by queens and workers throughout the colony cycle. While wax is biologically critical to colony growth, it is known that it can serve as a reservoir for pathogens and environmental contaminants in honey bee colonies (Flores et al. 2005; Fries 1988; Shimanuki & Knox 2000; Wu et al. 2011). The degree to which this is a problem in bumble bee colonies is not well understood and we consider this an area of severe data deficiency. However, as wax is not reused in production facilities, it poses little risk for horizontal transfer of pathogens in commercial bumble bees and thus is a low priority for study. However, we acknowledge that wax will remain in nest boxes that have been disposed of and may represent a source of infectivity after the colony is no longer in production. Proper cleaning and/or disposal of used equipment should mitigate any risks of wax vectoring disease or introducing environmental contaminants in rearing facilities. Researchers using nest boxes to trap wild queens in the field should be aware of the potential for contamination from previous nests if the boxes are re-used, and should consider decontamination treatments such as soaking material in a 10% bleach solution, or a period of exposure to UV radiation.

A summary table of parasitism, target (*i.e.,* adults, brood, pollen stores, nest material, etc.), incidence, threat imposed, and detection for the taxa here described (when known) can be found in the online Summary Table 1.

## Discussion

As we have pointed out elsewhere (Figueroa et al. 2023), many of the symbionts that are now affecting bumble bees have moved from another species of managed social bees, the honey bees (*Apis mellifera*). International trade in that species has also resulted in global movements of their parasites and other symbionts, and it’s not surprising that given the close contact of bees sharing floral resources, where disease transmission can occur (Adler et al. 2018), and the potential proximity of colonies of the two genera, that inter-specific transfer of some of these symbionts has occurred. Although much is now known about the diversity of bumble bee symbionts, as we have pointed out, there are still some important knowledge gaps to be filled, and the opportunity for new technology to be used in identifying them.

Commercialization of bumble bees as crop pollinators has required the development of large facilities where many bees are in close proximity. This creates opportunities for increased transmission of symbionts, and for movement of infected colonies as part of the international trade created by demand for pollinators. The use of pollen collected by honey bees to feed bumble bee larvae is another avenue for transfer of pathogens between the species. Perhaps the good news is that we now have a growing body of literature about the symbionts that are associated with both honey bees and bumble bees, that we have methods for detecting and identifying them, and can therefore manage the bees in ways that can reduce transmission. The severity of the negative effects of some of these symbionts, and the fact that they can be transmitted from managed bees to wild bees (Colla et al. 2006) provide incentive for development of management techniques and policies that will minimize future problems for this important group of pollinators (Strange et al. 2023).

## Supplementary Material

Supplement1
