# Supplementary material for: Parasites, parasitoids, and hive products that are potentially deleterious to wild and commercially raised bumble bees (Bombus spp.) in North America
Source: J Pollinat Ecol. Author manuscript; Available in PMC 2025 Jan 2. (PMC11694831; doi:10.26786/1920-7603(2023)710)
Supplement: Supplement1 [file NIHMS2029941-supplement-Supplement1.pdf]

DOI: 10.26786/1920-7603(2023)710

Order	Family	Genus	species	Mode of parasitism	Target	Incidence	Threat	Detection	Citation
**Acarina**			at least 91 species of mites	ectoparasites	adults				
	Gaudiellidae	*Cerophagus*	multiple species			rare?	low	visual	O'Connor 1992
	Acaridae	*Kunzia*	multiple species			rare?	low?	visual	Goldblatt & Fell 1984
	Podapolipidae	*Locustacarus*	*buchneri*	parasite of adults	adults	variable, but can be widespread	considered benign, but largely unknown		reviewed in Schmid-Hempel 1998; Otterstatter and Whidden 2004; Yoneda et al. 2008a, b; Arismendi et al. 2016
	Parasitidae	*Parasitellus (formerly Parasitus)*	multiple species	kleptoparasites	adults	rare?	low	visual	Eickwort 1990
	Melicharidae	*Proctolaelaps*	*bombophilus*	kleptoparasites	adults	rare?	low	visual	Klimov et al. 2016
			longisetosus	kleptoparasites	adults	rare?	low	visual	Haas et al. 2019
**Diptera**									
	Phoridae	*Apocephalus*	*borealis*	parasitoids	adults	rare?	Potentially high; can be fatal and could vector pathogens	visual on dissection, or rearing	Brown 1993, Otterstatter et al. 2002; Core et al. 2012
	Conopidae	*Physocephala*	at least 5 species	parasitoids	adults	Variable but can be high	Model predicted reduction in colony performance, especially under low resources	visual on dissection, or rearing	Freeman 1966; Camras 1996; Abdalla et al. 2014; Malfi et al. 2014; Gibson et al. 2016; Malfi et al. 2018
		Zodion				potentially 2 records from Canada (unverified); rare	low		MacFarlane and Pengelly 1974
	Sarcophagidae			may be primarily scavengers in nests					
		Boettcheria	*litorosa*	parasite of adults	adults	low	low	visual on dissection, or rearing	Ryckman 1953

		Liosarcophaga	*sarracenioides*	parasite of adults	adults	low	low	visual on dissection, or rearing	Ryckman 1953
		Brachicoma	*sarcophagina*	brood parasite	larvae, pupae	low	potentially severe	visual on dissection, or rearing	Macfarlane and Pengelley 1974, 1977
		Helicobia	*morionella*	parasite of adults	adults	low	low	visual on dissection, or rearing	Ryckman 1953
	Syrphidae								
		Volucella	*bombylans*	scavengers on nest debris		low	Other species of *Volucella* are parasites in wasp larvae	visual	reviewed in Schmid-Hempel 1998; Monfared 2013
**Hymenoptera**									
	Braconidae								
		Syntretus	*splendidus*	parasitoid	parasites of queens, males, workers	rare	minor (though possibly severe to queens); none to commercial bees	dissection, or rearing from infected adults	Alford 1968; reviewed in Schmid-Hempel 1998
	Eulophidae						potentially severe		González and Matthews 2005
		Melittobia	*acasta*	ectoparasitoid	prepupae, pupae	widespread	minor?	dissection, or rearing from infected adults	Matthews et al. 2009; Gekière et al. 2022
			australica	ectoparasitoid	prepupae, pupae	rare?	minor?	dissection, or rearing from infected adults	Matthews et al. 2009
			chalybii (possible mis-identification)	ectoparasitoid	prepupae, pupae	rare	minor?		Macfarlane & Pengerly 1977 (though Gonzales & Matthews 2005 note possible misidentification of M. acasta); Whitfield & Cameron 1993

	Apidae	*Bombus (Psithyrus)*		brood parasite	queens	widespread	none to commercial bees	observation of colony	Williams 2008
**Coleoptera**									
	Nitidulidae	*Aethina*	*tumida*	scavenger, predator	stores, eggs, larvae	rare	minor	observation of colony	Ambrose et al. 2000; Spiewok & Neumann
	Cryptophagidae	*Antherophagus*	*?*	phoretic, scavenger	nest detritus		minor	observation of colony	Bousquet 1989
	Cleridae	*Trichodes*	*ornatus*	predator	larvae, pupae	rare	minor	observation of colony	Hobbs et al. 1962
**Lepidoptera**									
	Pyralidae	*Aphomia*	*sociella*	scavenger, predator	wax, pollen, larvae	rare?	potentially severe	observation of colony	Solis and Metz 2008; Gekière et al. 2022
		Galleria	*mellonella*	scavenger		rare	minor?	observation of colony	Oertel 1963
		Vitula	*edmandsii*	scavenger	wax, pollen	widespread	minor?	observation of colony	Milum 1953; Ryckman 1953; Whitfield &
		Achroia	*grisella*	scavenger, predator		rare	pest of honey bee hives, potential for *Bombus* (not reported)	observation of colony	Milum 1940
		Plodia	*interpunctella*	scavenger		rare	potentially severe	observation of colony	An et al. 2007
